# Exploring Women’s Beliefs and Perceptions About Healthy Eating Blogs: A Qualitative Study

**DOI:** 10.2196/jmir.3504

**Published:** 2015-04-08

**Authors:** Véronique Bissonnette-Maheux, Veronique Provencher, Annie Lapointe, Marilyn Dugrenier, Audrée-Anne Dumas, Pierre Pluye, Sharon Straus, Marie-Pierre Gagnon, Sophie Desroches

**Affiliations:** ^1^Institute of Nutrition and Functional FoodsLaval UniversityQuebec, QCCanada; ^2^Department of Family MedicineMcGill UniversityMontreal, QCCanada; ^3^Department of Geriatric MedicineToronto UniversityToronto, ONCanada; ^4^Li Ka Shing Knowledge InstituteSt. Michael’s HospitalToronto, ONCanada; ^5^CHU de Québec Research CenterPopulation Health and Optimal Health Practices Research UnitQuebec, QCCanada; ^6^Faculty of NursingLaval UniversityQuebec, QCCanada

**Keywords:** blog, health behavior, nutrition, qualitative research, social media, knowledge translation

## Abstract

**Background:**

Chronic diseases are the leading cause of death (63%) worldwide. A key behavioral risk factor is unhealthy eating. New strategies must be identified and evaluated to improve dietary habits. Social media, such as blogs, represent a unique opportunity for improving knowledge translation in health care through interactive communication between health consumers and health professionals. Despite the proliferation of food and lifestyle blogs, no research has been devoted to understanding potential blog readers’ perceptions of healthy eating blogs written by dietitians.

**Objective:**

To identify women’s salient beliefs and perceptions regarding the use of healthy eating blogs written by dietitians promoting the improvement of their dietary habits.

**Methods:**

We conducted a qualitative study with female Internet users living in the Quebec City, QC, area with suboptimal dietary habits. First, the women explored 4 existing healthy eating blogs written in French by qualified dietitians. At a focus group 2-4 weeks later, they were asked to discuss their experience and perceptions. Focus group participants were grouped by age (18-34, 35-54, and 55-75 years) and by their use of social media (users/nonusers). Using a questionnaire based on the Theory of Planned Behavior, participants were asked to identify salient beliefs underlying their attitudes (advantages/disadvantages), subjective norms (what people important to them would think), and perceptions of control (facilitators/barriers) regarding the use of a healthy eating blog written by a dietitian to improve dietary habits. Discussion groups were audiotaped, transcribed verbatim, coded, and a deductive content analysis was performed independently by 2 individuals using the NVivo software (version 10).

**Results:**

All participants (N=33) were Caucasian women aged between 22 to 73 year. Main advantages perceived of using healthy eating blogs written by a dietitian were that they provided useful recipe ideas, improved lifestyle, were a credible source of information, and allowed interaction with a dietitian. Disadvantages included increased time spent on the Internet and guilt if recommendations were not followed. Important people who would approve were family, colleagues, and friends. Important people who could disapprove were family and doctors. Main facilitators were visually attractive blogs, receiving an email notification about new posts, and finding new information on the blog. Main barriers were too much text, advertising on the blog, and lack of time.

**Conclusions:**

The women in this study valued the credibility of healthy eating blogs written by dietitians and the contact with dietitians they provided. Identifying salient beliefs underlying women’s perceptions of using such blogs provides an empirically supported basis for the design of knowledge translation interventions to help prevent chronic diseases.

## Introduction

### Background

 Chronic diseases are long-lasting diseases that can be controlled but not cured [1]. They are the leading cause of death (63%) worldwide [2]. In Canada, the proportion of people dying from chronic conditions in 2010 was 89% [2]. The World Health Organization has projected that chronic disease deaths will have increased by 15% globally between 2010 and 2020 [3]. Most countries cannot combat chronic disease with medical care alone: prevention is essential [4]. Healthy eating is recognized as a critical factor in preventing many chronic health conditions, including hypertension, diabetes, cardiovascular diseases, and obesity [5,6]. Food skills, including nutrition knowledge and cooking skills [7], appear to be key to improving eating habits [8] and can help reduce the prevalence of obesity and other chronic diseases in the population [9]. Health communication is an effective strategy for changing norms and beliefs about dietary behaviors, especially by promoting knowledge about appropriate dietary choices [10]. Registered dietitians (RDs) are health care professionals who are trained to transfer this knowledge.

With the advent of Web 2.0, the Web has evolved toward greater simplicity and interactivity. Social media tools are increasingly being used for health communication [11-13], including to deliver health behavior change interventions [14-18], because they reach a large Internet population with diverse sociodemographic characteristics, independently of education, race/ethnicity, or health care access [19]. In the field of physical activity and nutrition, studies have shown promising results [20-26]. A recent literature review published in 2014 that included 22 randomized controlled trials found a significant decrease in dietary fat consumption with the use of social media promoting healthy diet and exercise in the general population [20]. Neuenschwander et al [27] and Bensley et al [28] found that Web-based nutrition education interventions can lead to favorable and effective changes in eating habits when compared with in-person nutrition education interventions. Although these studies show great potential for interventions that use the Internet, none of them included blogs. Blogs are discussion or information sites published by individuals or organizations on the Internet consisting of discrete entries, or posts, displayed in reverse chronological order (the most recent post appears first). Most blogs allow visitors to leave comments and message one another [29].

Studies conducted on health blogs [30-36] and food blogs [37-40] have focused primarily on identifying why bloggers write the blogs and on their information content rather than on blog readers’ health behaviors and outcomes. In addition, the majority of these studies refer to food blogs written by lay people and not by RDs. Discerning users have to sift through thousands of blogs to find credible information because noncredible, erroneous, or even harmful health information is widely available and may be increasing eating disorder behaviors [35,36].

To the best of our knowledge, no studies have examined users’ perceptions of healthy eating blogs written by RDs. They represent a unique opportunity for improving knowledge translation in nutrition through interactive communication between consumers and dietitians. However, to make use of blog technology to communicate healthy eating information, RDs must better understand how Internet users perceive healthy eating blogs written by qualified nutrition professionals.

*Healthy living blogs* have been defined as personal webpages devoted to sharing an individual’s healthy lifestyle for the purpose of providing an example of “healthy living” to others [35]. There is no standard definition of a healthy eating blog written by an RD; therefore, for the purpose of this study, we defined it as an interactive webpage written by a dietitian promoting the overall improvement of dietary habits (not focused on a specific diet-related disease).

The majority of food bloggers are women [37,39] and their readership is also mostly women who use blogs as an information source about food in general, recipes, or food preparation [41]. Although men also participate in meal preparation, women remain primarily responsible for food purchase and preparation in most households [42,43]. Consequently, health promotion strategies targeting women have the potential to impact not only women blog readers, but also their families.

### Purpose

The objective of this qualitative study was to identify the salient beliefs and perceptions of female users/potential users of blogs with suboptimal dietary habits regarding their intention to use a healthy eating blog written by an RD promoting the overall improvement of dietary habits.

### Theoretical Framework

A recent systematic review showed that Internet-based interventions developed using a theoretical foundation were more likely to predict behavior than atheoretical interventions [44]. Thus, to maximize the identification of beliefs related to the use of a healthy eating blog written by an RD, we based our focus group moderator’s guide and our qualitative analysis on the Theory of Planned Behavior (TPB), which identifies determinants of intention and behavior [45]. We chose this theory because several meta-analytic studies and systematic reviews have shown the strength of its constructs in predicting behavior in a wide variety of contexts [46,47], including healthy eating [48] and, more recently, the use of new Web 2.0 technologies [49-51]. In addition, studies on the adoption of new technologies have suggested a positive correlation between the perception of a blog’s usefulness and ease of use and the perceived attitude toward it [52,53].

**Figure 1:**
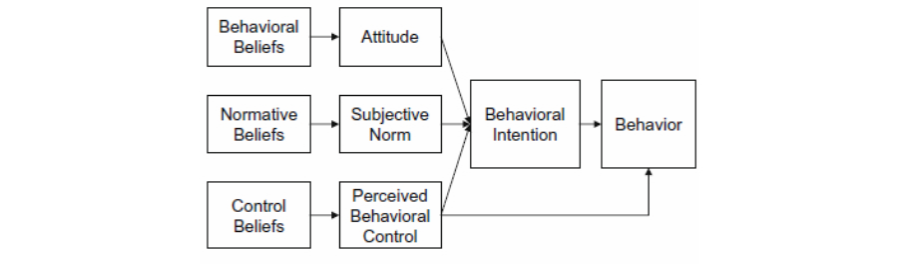
Ajzen’s Theory of Planned Behavior [45].

According to the TPB, intention to use a healthy eating blog written by an RD to improve dietary habits is determined by 3 factors (Figure 1): (1) behavioral beliefs, or underlying attitudes (advantages/disadvantages), toward the use of the blog; (2) normative beliefs, or underlying perceptions of whether important people in one’s life would approve or disapprove of the behavior; and (3) control beliefs, or underlying perception of facilitators and barriers to adopting the behavior. Thus, someone will be more inclined to use a healthy eating blog written by an RD to improve his/her dietary habits if he/she perceives more advantages and facilitators than disadvantages and barriers for doing so, and if people who are important to him/her would approve rather than disapprove of the behavior. Developing this knowledge base of beliefs underlying Internet users’ attitudes, subjective norms, and perceptions of control regarding healthy eating blogs written by an RD as a means to improve dietary habits will contribute to a better understanding of factors related to the intentions to use and use of these blogs that can contribute to the development of nutritional interventions.

## Methods

### Participants and Recruitment

We sought female participants with suboptimal dietary habits who were users or potential users of healthy eating blogs. Participants were recruited using the mailing list of the Institute on Nutrition and Functional Foods at Laval University, Quebec, QC, and ads in local newspapers. A total of 57 women responded to our recruitment call, among whom 5 did not meet our inclusion criteria and 19 were no longer interested after they received full information about the nature of their participation. Inclusion criteria were (1) to be a woman living in the Quebec City metropolitan area aged ≥18 years, (2) to have access to the Internet, (3) to use the Internet more than once a week, and (4) to consume 5 or less portions of fruit or vegetables per day (an RD assessed participant’s fruit and vegetable consumption over the past 24 hours). Fruit and vegetable consumption is considered a good predictor of overall diet quality [54]. Participants received no honorarium and all gave written informed consent. This project was approved by the Ethics Committee of Laval University (2012-204 A-1/18-03-2013).

### Data Collection Procedure

Participants were first interviewed individually and then invited to participate in 1 of 6 focus groups. The focus groups included 4 to 6 women each and were conducted between April and June 2013, 2 to 4 weeks following the individual interview. All focus groups were audiotaped and transcribed verbatim.

During the individual interview, the 33 women were first invited to complete an online sociodemographic questionnaire including 29 questions about their Internet use; the frequency with which they surfed the Internet, read, or commented on blogs; and if they ever looked for nutritional information online. Questionnaires were validated with 8 women to confirm the clarity of the questions prior to this study. Then, semistructured individual interviews were conducted face-to-face with a research coordinator and scheduled to last approximately 1 hour. This face-to-face interview was devoted to the exploration of 4 popular blogs written in French by RDs that promoted healthy eating. The 4 blogs were the same for all the participants and were chosen before the interview. To identify the 4 blogs, we used the Google search engine and typed the following French keywords: blog, *blogue* and *nutritionniste* or *diététiste* (dietitian). Blogs were included if they met the following criteria: (1) written in French by a Canadian RD, (2) targeted human nutrition as the unique topic, (3) had as its main objective the overall promotion of a healthy diet (ie, did not focus on a specific diet-related disease), and (4) was proactive (ie, had published a new post at least once a month since the creation of the blog and had published a minimum of 12 posts). During the interview, participants were asked their perceptions about design features and nutritional content of those 4 blogs to explore different facets characterizing existent healthy eating blogs written by dietitians. Because some women were not frequent users of blogs or other social media, the primary goal of the interview was to prepare the women to discuss their perceptions of healthy eating blogs written by an RD at the subsequent focus group.

Between 2 and 4 weeks after the individual interviews, the 33 women were invited to participate in a focus group of 90 minutes moderated by a trained research coordinator and an assistant (VBM). The moderator used a semistructured interview to ask participants questions about their perceptions of consulting healthy eating blogs written by RDs to promote improvement of dietary habits, and the assistant took notes during the discussion. The procedure and interview guide were based on Patton’s recommendations [55]. The standardized open-ended interview questionnaire was developed according to the 3 constructs of the TPB. Questions aimed to identify the salient beliefs underlying their attitudes (advantages/disadvantages), subjective norms (approval or disapproval of important others), and perception of control (facilitators/barriers) with regard to the use of blogs written by RDs promoting improvement of dietary habits, such as those they had encountered during the individual interviews. All women in each focus group were asked the same questions in the same order to increase comparability of responses. The interview guide was validated with a focus group of 6 women before conducting the experimental focus groups [55].

Six focus groups (4 to 6 women in each focus group) were needed to achieve theoretical saturation [56]. To determine saturation, we calculated the extent to which different focus groups mentioned the same themes. By the end of the fifth focus group, 98.6% of themes had been mentioned at least once; the remaining 1.4% of themes were only mentioned in the sixth focus group. Participants were grouped by age and by their use of social media (Facebook, Twitter, or blogs) to increase homogeneity and better describe population subgroups as per recommendations by Patton and collaborators [55]. Two focus groups included women aged between 18 and 34 years who used social media, 2 focus groups included women between 35 and 54 years who used social media, and 2 focus groups included women older than 55 years who did not use social media on a regular basis.

### Data Analysis

Descriptive statistics and mean ± SD were used to analyze all quantitative data from the online sociodemographic questionnaire using the SAS version 9.3 (SAS Institute, Inc, Cary, NC, USA). The steps of the deductive content analysis described by Elo et al [57] inspired the content analysis of the focus groups, which were transcribed verbatim. Quotes were coded line by line to bring out the main salient beliefs according to the construct of the TPB: behavioral beliefs, normative beliefs, and control beliefs (Figure 1). Two coders (VBM and MD) performed the coding with NVivo version 10 (QSR International, Cambridge, MA, USA) independently and then compared themes to reach consensus on the terminology to be used for each. A third person (AAD) validated the resulting categories and was available to resolve any discrepancies. For the purpose of our study, all beliefs that emerged in at least 2 groups of 6 (33%) were considered salient beliefs [58]. If the same belief was named by several participants in the same group, it was considered a single belief. Finally, categories obtained for groups according to participants’ age were compared to see if any age-related differences in salient beliefs emerged.

## Results

**Figure 2:**
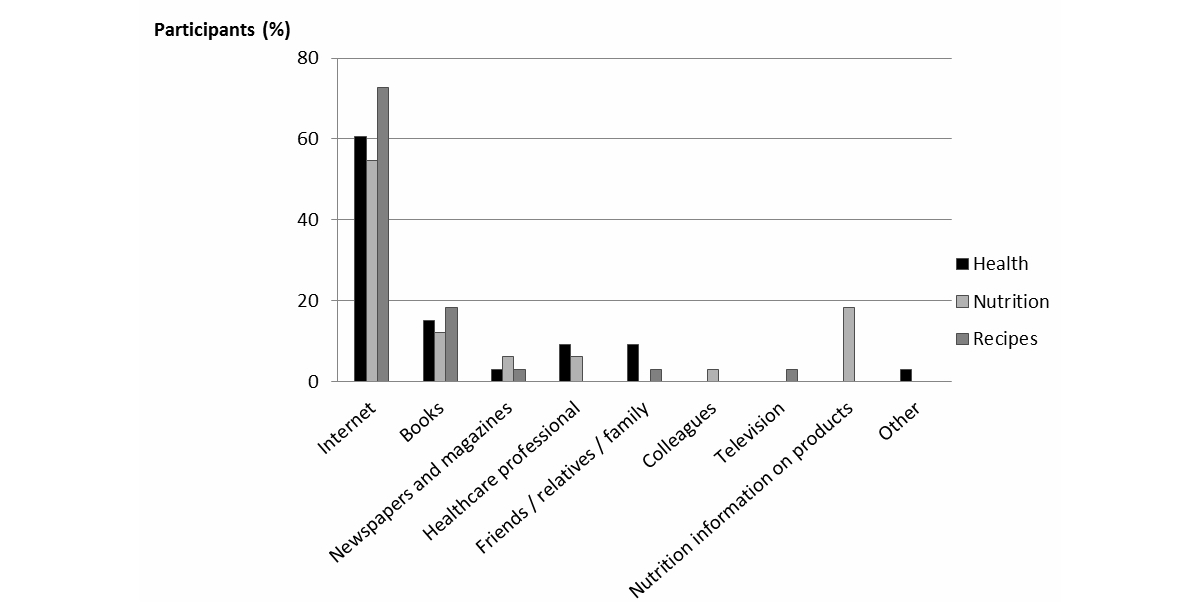
Information sources for information on health, nutrition, and recipes.

Of the 33 women who participated in the individual interviews, 29 also participated in 1 of the focus groups. The 4 women who did not complete the project mentioned lack of time or unforeseen personal circumstances on the date scheduled for the focus group. All participants were Caucasian females between 22 and 73 years of age, most were fairly highly educated and of above-average income (Table 1). Of the 33 women, 25 (76%) had consulted a blog before, but only 3 spent more than 10 hours a week on the Internet (Table 2). Yet the majority identified the Internet as their principal source of information about health, nutrition, and recipes (Figure 2).

Results of the analysis of the 6 focus groups are presented in Table 3, showing the women’s salient beliefs reported in each category and the frequency of mentions according to age group. Select quotes presented subsequently illustrate our key findings. Quotes were originally in French and have been translated by a professional translator.

**Table 1:** Sociodemographic characteristics of the 33 participants.

Characteristics		Participants
Age (years), mean (SD)		44 (17)
**Ethnicity, n (%)**		
	Caucasian	33 (100)
**Highest level of education completed, n (%)**		
	Primary	1 (3)
	High school	3 (9)
	College	13 (39)
	University	16 (48)
**Family income (Can $), n (%)**		
	0-19,999	4 (12)
	20,000-49,999	10 (30)
	50,000-99,999	8 (24)
	100,000-149,999	4 (12)
	150,000-199,999	5 (15)
	≥200,000	0 (0)
	Unknown	2 (6)

**Table 2:** Internet use characteristics of the 33 participants.

Internet characteristics		n (%)
**Time spent on Internet for leisure**		
	1-3 hours/week	13 (39)
	3-5 hours/week	6 (18)
	5-10 hours/week	11 (33)
	≥10 hours/week	3 (9)
Read a blog before		25 (76)
Read a blog on nutrition before^a^		16 (64)
Read comments on a blog before^a^		18 (72)
Commented on a blog before^a^		2 (8)

^a^Only among participants who had already read a blog before (n=25).

**Table 3:** Salient beliefs associated with the use of a healthy eating blog.

Salient beliefs		Frequency of groups, n (n=6)	Frequency of mentions by age (years), n		
			18-34 (n=2)	35-54 (n=2)	55-75 (n=2)
**Behavioral beliefs-perceived advantage**					
	Gives recipe ideas	5	2	2	1
	Helps improve diet-related lifestyle	4	1	1	2
	Promotes interaction with the dietitian	4	2	0	2
	Interesting to have the opinion of fellow blog readers	3	1	2	0
	Introduces to new foods	3	1	0	2
	Introduces to new trends in nutrition	3	1	1	1
	Allows to learn more about nutrition in general	3	0	2	1
	Credible information	3	1	0	2
	Allows to have the opinion of a dietitian	2	0	1	1
**Behavioral beliefs-perceived disadvantages**					
	Opinion of only 1 blogger (if only 1 blogger is followed)	2	2	0	0
	Increases time spent on the Internet	2	1	1	0
	Nonrelevant comments from blog readers	2	1	1	0
	Feeling of guilt if one does not follow dietary advice given on the blog	2	1	1	0
**Normative beliefs-approval**					
	Family	5	2	1	2
	Colleagues	5	2	2	1
	Friends	4	2	1	1
	Physicians	2	0	0	2
	Neighbors	2	0	1	1
**Normative beliefs-disapproval**					
	Family	4	2	2	0
	Physicians	3	0	1	2
	Friends	2	1	0	1
	Colleagues	2	1	1	0
**Control beliefs-facilitators**					
	Visually attractive blog	6	2	2	2
	Email to inform of a newly published posts	5	1	2	2
	New information weekly (updated weekly)	5	1	2	2
	Well-designed for quick access to desired information	4	0	2	2
	Interesting and entertaining posts	4	2	2	0
	Quick access to the blog	3	2	0	1
	Well written, concise info	3	1	1	1
	Written by a personality	3	1	1	1
	Notification through Facebook to inform of a newly published posts	3	2	1	0
	Presence of recipes	3	1	2	0
	Contains complete information	3	1	2	0
	Popular blog, referred by someone	3	1	1	1
	Text well-structured in paragraphs with subtitles	3	0	2	1
	Availability of nutritional value of the recipes	2	1	1	0
	Opportunity to read other readers’ comments and to link with them	2	1	1	0
	Possibility to ask questions, interactivity	2	0	1	1
	Easy to understand, not too scientific	2	0	1	1
	Narrative approach	2	0	2	0
	Posts about novel foods, trends	2	1	1	0
**Control beliefs-barriers**					
	Posts too long to read	5	1	2	2
	Presence of advertising	5	1	2	2
	Smaller font size	4	1	2	1
	Conflict of interests, promotion of commercial products by blogger	4	1	2	1
	Lack of new information, too repetitive	4	1	1	2
	Lack of time	4	1	1	2
	Poorly structured, complicated site	4	1	2	1
	Notifications too frequent	4	2	1	1
	More negative than positive posts	3	1	1	1
	Visually unattractive	3	0	2	1
	Computer issues (eg, slow)	2	1	0	1
	Information not available, not accessible	2	0	1	1
	Credibility: can be written by anyone, not always true	2	1	1	0
	If it is not professional, poor writing	2	0	2	0

### Behavioral Beliefs: Perceived Advantages and Disadvantages

The most frequently cited advantages women perceived to consulting a healthy eating blog by an RD were that it gives recipe ideas (5/6 groups, 83%), helps improve diet-related lifestyle (4/6 groups, 67%), and promotes interaction with a dietitian (4/6 groups, 67%):

What’s useful with following a blog is if you can comment, you create a link with the nutritionist and you can follow her, and so there’s a connection that builds up.

group #3, participant 21

Numerous participants aged 35 years and older considered healthy eating blogs as a tool for learning more about nutrition in general (3/4 groups, 75%):

*I mean food is such a huge topic, it’s impossible to know everything about it, so you can go and look at what you like* [on the blog] *and that leads you on to new ideas for trying new things out*. (group #1, participant 3)

The 4 most cited disadvantages were having the opinion of only 1 dietitian-blogger (if only 1 blog was followed) (2/6 groups, 33%), increased time spent on the Internet (2/6 groups, 33%), irrelevant comments from other readers on the blog (2/6 groups, 33%), and guilt arising from not complying with the dietary recommendations on the blog (2/6 groups, 33%):

If you’re working...sometimes, I don’t even have time to stop and eat at work, or whatever. So then, I go look at it [the blog], and there are some great recipes. I don’t have time to go grocery shopping, I don’t have time to make them. And then, ah, I’m so discouraged

group #3, participant 20

Although groups comprised of women ≥55 years mentioned few disadvantages (n=2) of consulting a healthy eating blog by an RD, each was only mentioned in 1 group.

### Normative Beliefs: Approval and Disapproval

Participants identified many individuals they believed would approve of them reading a blog to improve their dietary habits. Among them, family (5/6 groups, 83%), colleagues (5/6 groups, 83%), and friends (4/6 groups, 67%) were most frequently mentioned as normative referents. Interestingly, most participants (4/6 groups, 67%) considered reading and interacting with a blog as a personal or a private action, so it was unclear to them (and perhaps irrelevant) whether people would approve or disapprove:

The things I go and look at on the Internet, they’re also personal. I’m not the type to go talking about everything I do. So it’s not likely that I’ll know if people around me are against it—I keep it to myself. If I’m interested in nutrition, in going on blogs, that’s my business, it’s not anyone else’s.

group #5, participant 33

Almost all groups (5/6 groups, 83%) reported that it was difficult to be against healthy eating considering that it is an inoffensive subject. However, some participants identified their family (4/6 groups, 67%) and physicians (3/6 groups, 50%) as the persons most likely to disapprove of their use of blogs to improve dietary behaviors, perhaps because of erroneous or harmful information that can be found on health blogs:

My doctor already warned me, because I’ve told her twice that I go on it. She said for sure there are some good things, but she said to be careful. She meant that not everything out there is good.

group #2, participant 2

### Control Beliefs: Facilitators and Barriers

All groups mentioned that visual characteristics of the blog were important facilitators:

An attractive site, that helps too. When it’s just text with no pictures, it’s heavy. I’m very...I like it when it’s simple, very visual, that makes me want to go look at it.

group #3, participant 29

Most women said that facilitators were receiving an email notifying them of a new post published on the blog (5/6 groups, 83%) and having new content or new information each week (5/6 groups, 83%):

It shouldn’t always repeat the same thing. You can get tired of it, you know. It has to be a little different ... for example, every week something new that will make us want to go check it out

group #2, participant 2

Interesting and entertaining posts as well as well-designed blogs allowing quick access to the desired information were also identified frequently as facilitators (4/6 groups, 67%).

In contrast, the most often reported barriers to consulting a healthy eating blog by an RD were posts being too long (5/6 groups, 83%), presence of advertising (5/6 groups, 83%), bloggers being in conflict of interest by promoting products on their blog (4/6 groups, 67%), and lack of time (4/6 groups, 67%).

Lack of new information, repetitive posts, small font sizes, or badly structured sites were also considered barriers to using a blog by most women (4/6 groups, 67%). Finally, receiving notifications of new posts too often was also identified as a barrier (4/6 groups, 67%):

If it’s irritating, if someone or something doesn’t stop bugging me, I’m gonna go, like, ok, this is junk mail.

group #3, participant 8

## Discussion

### Principal Findings

Our objective in conducting this qualitative study was to better understand women’s perceptions toward healthy eating blogs written by RDs as a tool to improve their dietary habits. Identifying the salient beliefs underlying women’s attitudes, subjective norms, and perceptions of control have important implications for developing interventions to prevent diet-related chronic diseases, including helping dietitians to design user-friendly and relevant healthy eating blogs to improve dietary habits and to assess their efficacy. Many of our results can be read as clear design guidelines regarding details such as blog length, website structure, email notifications, and visual attractiveness. In addition, we believe the following 5 points are worthy of further discussion.

First, results of our study show that healthy eating blogs written by RDs may be an important nutritional knowledge translation tool for preventing chronic disease. The women perceived numerous advantages, including increased knowledge about new foods, nutrition trends, healthy recipes, and knowledge about nutrition in general, and they identified few disadvantages. This is coherent with findings by Edwards et al [59] who reported that among chronic disease patients, the most important constructs related to interest in use of technology to provide health care remotely were (1) confidence in using it and (2) perceiving greater advantages and fewer disadvantages.

Second, the credibility of the blogger and the information found on blogs were not barriers mentioned frequently by participants in our study. Given that many studies have found that credibility is a concern frequently mentioned by users searching for health information on social media [60,61], this suggests that the fact that the blogs in our study were written by RDs made a difference to the participants’ confidence in them. This suggestion is upheld by Greenberg et al [57] who reported that blogs written by experts are perceived as being more credible than personal blogs. However, the extent to which food blogs not written by accredited health care professionals such as dietitians contain erroneous information remains to be studied. In a recent survey of 679 food bloggers, 87% said they were writing a blog primarily because of their passion for food and only 22% had a work history relating to food (not necessarily dietitians) [62]. The credibility of information found on blogs is thus an important concern not only for health care providers, but also for consumers of healthy eating blogs and suggests that there might be an important role for dietitians in designing interventions that will be of interest to consumers.

Third, an interesting feature of blogs as potential tools for promoting better health is their interactive nature [63]. Many women in our study perceived that interacting with others, including the other blog users but especially with an expert dietitian-blogger, was one of the chief advantages of consulting healthy eating blogs written by RDs. Recent studies have shown that increased interaction, shared information, and peer support are key benefits to using social media for health communication [52,53]. In contexts where the health care system provides limited access to live health care professionals, this feature may be especially attractive to potential users.

Fourth, our results show that healthy eating blogs written by a dietitian may not yet be the media of choice for translating nutritional knowledge to older women. First, although they cited few disadvantages (determinant of attitude), they mentioned several barriers (determinant of perceived control), suggesting that their issues with blogs were more in the domain of perceived capacity. Also, the features they appreciated, such as email notifications about new posts rather than a notification through Facebook for instance, suggest that these women may be more comfortable with email than with more recent interactive communication technologies (eg, Facebook). In our study, the nutrition-related content of the post (eg, recipes, quality of information) seemed more important for young women, whereas the site design (structure of the blog and the posts) seemed more important for older women. According to Chou and collaborators [19], age is the greatest predictor of the use of social media, with a lower penetration in the older population aged 55 years and older. However, studies also suggest that this phenomenon is changing. According to a recent Pew Internet survey, 50% of respondents aged 50-64 years reported using social media in 2012 compared to approximately 15% in 2008 [64]. Designers should include users of all ages in the process of developing different knowledge translation strategies.

Finally, our results raise the theoretical question of the use of the unmodified TPB for examining predictors of intention for using social media, particularly the relevance of the subjective norm construct. Women in our study considered that consulting a healthy eating blog by an RD to improve their dietary habits was a personal and individual action that did not necessarily require approval from others. Several cognitive behavior models that account for technology acceptance have been explored in the quest for a “unified theory” that may provide fruitful avenues for modifying or adding to the TPB in future studies of the experiences of potential users of healthy eating blogs written by RDs with a view to developing effective interventions for chronic disease prevention [51,52].

### Limitations

Godin et al [58] suggest that a sample of 25-30 participants is appropriate to highlight salient beliefs of a population, the participants in our study were all Caucasians, mostly educated above high school level (college and university), and had a relatively high family salary income; therefore, they are not fully representative of the general population. Second, this was an experimental and not a natural setting. However, as we wished to reach potential users as well as users, we were obliged to introduce potential users to blogs. In addition, we exercised control over the setting because we were exploring blogs specifically written by dietitians.

### Conclusion and Implications for Health Care

As the interest for the use of social media, such as blogs, in the population is growing, there is an urgent need to assess their impact on health outcomes. Research in blogging as a potential tool for knowledge translation in health is in its infancy and much work remains to be done before we can determine its effectiveness for improving healthy eating. Our study identified the perceptions of female Internet users with regard to their use of healthy eating blogs written by RDs. Our results can help design interventions that address attitudes, facilitators, and barriers in developing theory-based dietary behavior change interventions with a view to preventing diet-related chronic diseases.

## Abbreviations

RD: registered dietitian
TPB: Theory of Planned Behavior
